# Correction: Children's understanding of when a person's confidence and hesitancy is a cue to their credibility

**DOI:** 10.1371/journal.pone.0229410

**Published:** 2020-02-13

**Authors:** The PLOS ONE Staff

## Notice of republication

Incorrect versions of Figs 1, 2, 4, and 5 were published in error. The publisher apologizes for this error. This article was republished on February 4, 2020, to correct for this error. Please download this article again to view the correct version.
