# Supplementary material for: Children's understanding of when a person's confidence and hesitancy is a cue to their credibility
Source: PLoS One. 2020 Jan 27;15(1):e0227026. doi: 10.1371/journal.pone.0227026 (PMC6984727; doi:10.1371/journal.pone.0227026)
Supplement: S1 Table — (DOCX) [file pone.0227026.s001.docx]

**S1 Table. Experiment 1 Participant demographic information by age years.**

| Age Group | *N* | Age [*M* (*SD*)] | Female (%) |
| --- | --- | --- | --- |
| 3y - 3y11m | 85 | 3.49 (0.33) | 45.9 |
| 4y - 4y11m | 77 | 4.43 (0.26) | 54.5 |
| 5y - 5y11m | 71 | 5.47 (0.28) | 52.1 |
| 6y - 6y11m | 77 | 6.42 (0.29) | 46.8 |
| 7y - 7y11m | 66 | 7.38 (0.30) | 53 |
| 8y - 8y11m | 61 | 8.44 (0.31) | 45.9 |
| 9y - 9y11m | 20 | 9.47 (0.30) | 65 |
| 10y - 10y11m | 16 | 10.51 (0.34) | 62.5 |
| 11y - 11y11m | 15 | 11.38 (0.30) | 33.3 |
| 12y - 12y10m | 14 | 12.40 (0.29) | 50 |
