# Supplementary material for: Children's understanding of when a person's confidence and hesitancy is a cue to their credibility
Source: PLoS One. 2020 Jan 27;15(1):e0227026. doi: 10.1371/journal.pone.0227026 (PMC6984727; doi:10.1371/journal.pone.0227026)
Supplement: S2 Table — (DOCX) [file pone.0227026.s002.docx]

**S2 Table. Regression Analyses on Children’s Learning Preferences in Experiment 2 History Phase with Exclusions.**

|  | **Model 1** | | | **Model 2** | | |
| --- | --- | --- | --- | --- | --- | --- |
| *Predictors* | *Odds Ratios* | *CI* | *p* | *Odds Ratios* | *CI* | *p* |
| (Intercept) | 1.61 | 1.18 – 2.20 | **0.003** | 1.49 | 1.00 – 2.23 | 0.052 |
| Age (years, scaled) |  |  |  | 1.20 | 0.85 – 1.70 | 0.295 |
| Model Identity (1 = Andrea Knows) |  |  |  | 1.08 | 0.56 – 2.05 | 0.824 |
| Observations | 204 | | | 204 | | |
| *N* | 51 | | | 51 | | |
