# Supplementary material for: Children's understanding of when a person's confidence and hesitancy is a cue to their credibility
Source: PLoS One. 2020 Jan 27;15(1):e0227026. doi: 10.1371/journal.pone.0227026 (PMC6984727; doi:10.1371/journal.pone.0227026)
Supplement: S3 Table — (DOCX) [file pone.0227026.s003.docx]

**S3 Table. Regression Analyses on Children’s Learning Preferences in Experiment 2 Ask and Endorse Trials with Exclusions.**

|  | **Model 1** | | | **Model 2** | | |
| --- | --- | --- | --- | --- | --- | --- |
| *Predictors* | *Odds Ratios* | *CI* | *p* | *Odds Ratios* | *CI* | *p* |
| (Intercept) | 1.48 | 1.12 – 1.98 | **0.007** | 1.57 | 1.12 – 2.20 | **0.008** |
| Trial (1= Endorse) | 1.14 | 0.76 – 1.70 | 0.530 | 1.14 | 0.76 – 1.70 | 0.528 |
| Age (years, scaled) |  |  |  | 1.10 | 0.88 – 1.39 | 0.396 |
| Model Identity (1 = Andrea Knows) |  |  |  | 0.83 | 0.54 – 1.27 | 0.383 |
| Observations | 405 | | | 405 | | |
| *N* | 51 | | | 51 | | |
