# Supplementary material for: Children's understanding of when a person's confidence and hesitancy is a cue to their credibility
Source: PLoS One. 2020 Jan 27;15(1):e0227026. doi: 10.1371/journal.pone.0227026 (PMC6984727; doi:10.1371/journal.pone.0227026)
Supplement: S4 Table — (DOCX) [file pone.0227026.s004.docx]

**S4 Table. Regression Analyses on Children’s Learning Preferences in Experiment 3 History Phase with Exclusions.**

|  | **Model 1** | | | **Model 2** | | |
| --- | --- | --- | --- | --- | --- | --- |
| *Predictors* | *Odds Ratios* | *CI* | *p* | *Odds Ratios* | *CI* | *p* |
| (Intercept) | 1.36 | 1.03 – 1.81 | **0.029** | 1.60 | 1.05 – 2.45 | **0.029** |
| Age (years, scaled) |  |  |  | 1.33 | 0.99 – 1.78 | 0.055 |
| Model Identity (1 = Andrea Knows) |  |  |  | 0.73 | 0.41 – 1.30 | 0.288 |
| Observations | 201 | | | 201 | | |
| *N* | 51 | | | 51 | | |
