# Supplementary material for: Children's understanding of when a person's confidence and hesitancy is a cue to their credibility
Source: PLoS One. 2020 Jan 27;15(1):e0227026. doi: 10.1371/journal.pone.0227026 (PMC6984727; doi:10.1371/journal.pone.0227026)
Supplement: S5 Table — (DOCX) [file pone.0227026.s005.docx]

**S5 Table. Regression Analyses on Children’s Learning Preferences in Experiment 3 Ask and Endorse Trials with Exclusions.**

|  | **Model 1** | | | **Model 2** | | |
| --- | --- | --- | --- | --- | --- | --- |
| *Predictors* | *Odds Ratios* | *CI* | *p* | *Odds Ratios* | *CI* | *p* |
| (Intercept) | 1.08 | 0.82 – 1.42 | 0.580 | 0.97 | 0.69 – 1.37 | 0.881 |
| Trial (1= Endorse) | 0.97 | 0.66 – 1.43 | 0.889 | 0.97 | 0.66 – 1.44 | 0.896 |
| Age (years, scaled) |  |  |  | 1.11 | 0.91 – 1.36 | 0.291 |
| Model Identity (1 = Andrea Knows) |  |  |  | 1.20 | 0.81 – 1.78 | 0.367 |
| Observations | 411 | | | 411 | | |
| *N* | 52 | | | 52 | | |
