# Supplementary material for: Children's understanding of when a person's confidence and hesitancy is a cue to their credibility
Source: PLoS One. 2020 Jan 27;15(1):e0227026. doi: 10.1371/journal.pone.0227026 (PMC6984727; doi:10.1371/journal.pone.0227026)
Supplement: S6 Table — (DOCX) [file pone.0227026.s006.docx]

**S6 Table. Regression Analyses on Children’s Smartness Judgments in Experiment 3 with Exclusions.**

|  | **Model 1** | | | **Model 2** | | |
| --- | --- | --- | --- | --- | --- | --- |
| *Predictors* | *Odds Ratios* | *CI* | *p* | *Odds Ratios* | *CI* | *p* |
| (Intercept) | 1.36 | 0.79 – 2.36 | 0.269 | 0.51 | 0.21 – 1.22 | 0.129 |
| Age (years, scaled) |  |  |  | 1.56 | 0.81 – 3.01 | 0.180 |
| Model Identity (1 = Andrea Knows |  |  |  | 6.90 | 1.95 – 24.37 | **0.003** |
| *N* | 52 | | | 52 | | |
