# Supplementary material for: Children's understanding of when a person's confidence and hesitancy is a cue to their credibility
Source: PLoS One. 2020 Jan 27;15(1):e0227026. doi: 10.1371/journal.pone.0227026 (PMC6984727; doi:10.1371/journal.pone.0227026)
Supplement: S1 File — (DOCX) [file pone.0227026.s007.docx]

**S1 File. Regression Analyses on Children’s Learning Preferences in Experiment 1 Excluding Participants Who Failed the Manipulation Check.**

The following analyses were conducted as reported in the main text, but after excluding the (*N* = 47) participants who did *not* correctly answer the manipulation check post-test question (i.e., did not remember which model had seen inside the box during the History Phase). Our results reported in the main text do not change after making these exclusions.

**Preliminary analyses**

After exclusions, preliminary analyses indicated that there were no effects on learning preferences of participant sex, data collection location, model speaking order, or novel answers provided (all *ps* > .17), thus all subsequent analyses were collapsed across these variables. As in the main text, a significant main effect of the identity of the Confident model (*p* = .003) was found, and addressed below. Importantly there was no interaction between model identity and condition (*p* = .85).

**Who do children prefer to learn from?**

After excluding participants for failing the memory item, our results reported in the main text (i.e., without exclusions) are unchanged. Children’s responses (0 = Hesitant model; 1 = Confident model) to the four *Ask* and four *Endorse* trials were modeled simultaneously with a random-intercept logistic regression with participant ID as a random effect to account for the repeated responses using *lme4* [42] in R [43] (Table A). In Model 1, we find that children’s learning preferences vary by condition: In the Informed Condition the odds that children preferred to learn from the previously Confident model was 21% greater than from the previously Hesitant Model. This preference to learn from the more confident model is consistent with the results of earlier work showing that children, like adults, capitalize on the ‘confidence heuristic’ and expands on this earlier work in a number of important ways (refer to the ‘General Discussion’). In comparison, in the Uninformed Condition (*OR* = 0.82, .95CI = [0.72, 0.94], *p* = .004) the predicted odds of learning from the previously Confident model decreased by 18% compared to the Informed Condition.

**Table A. Regression Analyses on Children’s Learning Preferences in Experiment 1 with Exclusions**

|  | Model 1 | | Model 2 | |
| --- | --- | --- | --- | --- |
|  | *OR (.95%CI)* | *p* | *OR (.95%CI)* | *p* |
| Intercept | 1.21 (1.08 – 1.36) | .001 | 1.06 (0.93 – 1.22) | .379 |
| Condition  (1 = Uninformed) | 0.82 (0.72 – 0.94) | .004 | 0.82 (0.72 – 0.94) | .004 |
| Trial Type (1 = Endorse) | 1.02 (0.89 – 1.16) | .771 | 1.02 (0.89 – 1.17) | .756 |
| Age (Years, Scaled) |  |  | 1.04 (0.97 – 1.11) | .291 |
| Andrea Confident  (1 = Yes) |  |  | 1.27 (1.12 – 1.46) | <.001 |
| N_ID_ | 454 | | 454 | |
| Observations | 3572 | | 3572 | |

Importantly, the effect of condition was robust to the addition of control variables (Model 2). Even though both models were confident when making their statements during the Test Phases, children had a slight preference to favor the Hesitant model in the Uninformed Condition, suggesting they recognized that she was the better calibrated model. Finally, no clear developmental pattern emerged in children’s learning preferences in the regression model. However, post-hoc tests treating age categorically revealed that three- and four-year-olds did not differ in their tendency to favor the confident model between conditions: (Informed Condition: *M* = .54, *SD* = .22 versus Uninformed Condition: *M* = .51, *SD* = .22), *t* (113.62) = -.03, *p* = .409, *ns*. In contrast, both the 5- and 6-year-old group, and those age 7 and older, were less likely to choose the confident informant in the Uninformed condition (5-6 year olds *M* = .48, *SD* = .19; Ages 7+: *M* = .51, *SD* = .13) than in the Informed Condition (5-6 year olds *M* = .57, *SD* = .19, 7+: *M* = .55, *SD* = .18), *t* (125) = -2.544, *p* = .01 non-directional, and *t* (163.95) = -1.912, *p* = .055, directional, respectively.

**Who do children think is smarter?**

After excluding for failed memory tests, we again tested whether children differed between conditions in trait judgments of which model they thought was smarter (forced-choice). And our results reported in the main text remain unchanged. If children took into account a model’s calibration, we would expect children to judge the Confident model as smarter in the Informed Condition and the Hesitant model as smarter in the Uninformed Condition. We also tested whether their choice of who was smarter varied with age. We found that the odds of the confident model being judged as ‘smarter’ were 2.54 times greater (.95CI = [1.90, 3.40], *p* < .001) in the Informed Condition (see Table S2 – Model 1).

**Table B. Regression Analyses on Children’s Smartness Judgments in Experiment 1 with Exclusions**

| Outcome   (1 = Confident Model) | Model 1 | | | Model 2 | | Model 3 | |
| --- | --- | --- | --- | --- | --- | --- | --- |
|  | *OR (.95%CI)* | | *p* | *OR (.95%CI)* | *p* | *OR (.95%CI)* | *p* |
| Intercept | 2.54 (1.90 – 3.40) | <.001 | | 1.65 (1.16 – 2.33) | .005 | 1.67 (1.18 – 2.37) | .004 |
| Condition  (1 = Uninformed) | 0.46 (0.31 – 0.68) | <.001 | | 0.46 (0.31 – 0.69) | <.001 | 0.46 (0.30 – 0.69) | <.001 |
| Andrea Confident  (1 = Yes) |  | |  | 2.38 (1.59 – 3.55) | <.001 | 2.40 (1.60 – 3.59) | <.001 |
| Age (Years, scaled) |  | |  | 0.92 (0.75 – 1.12) | .386 | 1.18 (0.88 – 1.60) | .264 |
| Condition * Age |  | |  |  |  | 0.60 (0.40 – 0.91) | .017 |
| Observations | 444 | | | 444 | | 444 | |
| AIC | 574.57 | | | 559.48 | | 555.63 | |

Indeed, the odds that children thought the Confident model was smarter in the Uninformed condition were 54% less (.95CI = [0.31, 0.68], *p* < .001). This effect was robust to the addition of controls (Model 2). Importantly, this effect of condition was significantly moderated by age (Model 3). In the Uninformed Condition, the odds of judging the Confident model as ‘smarter’ significantly decreased with age (*OR* = 0.60, .95CI = [0.40, 0.91], *p* = .017).
