# Supplementary material for: Children's understanding of when a person's confidence and hesitancy is a cue to their credibility
Source: PLoS One. 2020 Jan 27;15(1):e0227026. doi: 10.1371/journal.pone.0227026 (PMC6984727; doi:10.1371/journal.pone.0227026)
Supplement: S2 File — (DOCX) [file pone.0227026.s008.docx]

**S2 File. Full Details of the Regression Analyses Comparing Experiments 2 and 3**

Post-hoc analyses were conducted to allow for a more direct comparison of children’s performance in Experiments 2 versus 3. Preliminary omnibus analyses indicated that preferences for learning from the calibrated model on the History, Ask, and Endorse trials were different between studies (*p* = .001), and order of Endorse and Ask trials (Trial Order, *p* = .04). This omnibus test did not reveal any detectable influence of model identity or its interaction with other variables (*p*s > .12). However, given the significant model identity effects in Experiment 1, we conservatively retained model identity as a covariate in the following models. We did not find that preferences were different across sex, speaking order, or which word set came first (*p*s > .18). Thus, we collapsed across these latter variables, but retain the others (Trial Order and Model Identity) in our models.

In Table A, we present the results of predicting preferences for learning on the Ask and Endorse trials from the calibrated model across studies in random-intercept logistic regression with participant ID as a random effect. The results of which reveal children’s clear preferences for the calibrated (informed and confident) model in Experiment 2 (Model 1 Intercept: *OR* = 1.36, .95CI = [1.12, 1.64], *p* = .002), but that singling out the well-calibrated model when both have been hesitant is considerably more difficult (Model 1 Study: *OR* = 0.64, .95CI = [0.52, 0.80], *p* < .001). Preferences for the calibrated model were not predicted by age. In an additional model, we found that age did not interact with experiment in predicting preferences. In Model 3, we find that preferences for the calibrated model were significantly reduced in Experiment 2 when Andrea was informed. However, the difference in preferences across studies was robust to the addition of control variables.

**Table A. Regression Analyses Comparing Learning Preferences on the Ask and Endorse test trials in Experiment 2 versus Experiment 3.**

|  | Model 1 | | Model 2 | | Model 3 | |
| --- | --- | --- | --- | --- | --- | --- |
|  | *OR (.95%CI)* | *p* | *OR (.95%CI)* | *p* | *OR (.95%CI)* | *p* |
| Intercept | 1.36  (1.12 – 1.64) | .002 | 1.37  (1.12 – 1.66) | **.002** | 1.62  (1.27 – 2.07) | **<.001** |
| Trial Type  (1 = Endorse) | 1.00  (0.80 – 1.25) | .975 | 1.00  (0.80 – 1.25) | .974 | 1.00  (0.80 – 1.25) | .980 |
| Study  (1 = Experiment 3) | 0.64  (0.52 – 0.80) | <.001 | 0.64  (0.50 – 0.81) | **<.001** | 0.63  (0.50 – 0.81) | **<.001** |
| Age  (years, scaled) |  |  | 1.02  (0.90 – 1.15) | .783 | 1.03  (0.91 – 1.16) | .635 |
| Trial Order  (1 = 2) |  |  |  |  | 0.90  (0.72 – 1.13) | .365 |
| Andrea Knows  (1 = Yes) |  |  |  |  | 0.78  (0.62 – 0.97) | **.029** |
| N_UniqueID_ | 160 | | 160 | | 160 | |
| Observations | 1268 | | 1268 | | 1268 | |
| AIC | 1747.516 | | 1749.440 | | 1747.634 | |

In Table B below, we present the results of predicting preferences for learning from the calibrated model on the History trials in random-intercept logistic regression with participant ID as a random effect. In the History phase of Experiment 2, participants clearly preferred the well-calibrated model (Model 1 Intercept: *OR* = 1.40, .95CI = [1.11, 1.77], *p* = .004). In comparison, the odds that preferences in Experiment 3 were for the calibrated model decreased by almost 50% (*OR* = 0.55, .95CI = [0.39, 0.77], *p* < .001), indicating that children were wisely choosing the visually-informed model on these trials. Although preferences for the calibrated model in the History phase across studies was not predicted by age, we find that older children in Experiment 3 increasingly preferred the miscalibrated (but visually-informed) model (*OR* = 0.72, .95CI = [0.55, 0.93], *p =* .01).

**Table B. Regression Analyses Comparing Learning Preferences on the History trials in Experiment 2 versus Experiment 3.**

|  | Model 1 | | Model 2 | | Model 3 | |
| --- | --- | --- | --- | --- | --- | --- |
|  | *OR (.95%CI)* | *p* | *OR (.95%CI)* | *p* | *OR (.95%CI)* | *p* |
| Intercept | 1.40  (1.11 – 1.77) | .004 | 1.60  (0.72 – 3.54) | .244 | 0.81  (0.32 – 2.09) | .669 |
| Study  (1 = Experiment 3) | 0.55  (0.39 – 0.77) | <.001 | 0.56  (0.39 – 0.81) | **.002** | 5.27  (0.87 – 31.86) | .070 |
| Age  (years, scaled) |  |  | 0.97  (0.86 – 1.10) | .634 | 1.08  (0.93 – 1.26) | .295 |
| Andrea Knows  (1 = Yes) |  |  | 1.12  (0.80 – 1.57) | .514 | 1.13  (0.81 – 1.57) | .482 |
| Study * Age |  |  |  |  | 0.72  (0.55 – 0.93) | **.013** |
| N_UniqueID_ | 159 | | 159 | | 159 | |
| Observations | 628 | | 628 | | 628 | |
| AIC | 862.179 | | 865.573 | | 861.445 | |

In Table C, we present the results of a series of logistic regression models predicting who children thought was ‘smarter’ in Experiments 2 and 3. We find that the odds of attributing the calibrated model with intelligence were vastly greater in Experiment 2 where both models were confident. However, in Experiment 3, participants were much more likely to think the non-calibrated, but *informed* model was smarter. Trait attributions of intelligence did not vary with age, and age did not interact with experiment.

**Table C. Regression Analyses Comparing Smartness Judgments on the History trials in Experiment 2 versus Experiment 3.**

|  | Model 1 | | Model 2 | | Model 3 | |
| --- | --- | --- | --- | --- | --- | --- |
|  | *OR (.95%CI)* | *p* | *OR (.95%CI)* | *p* | *OR (.95%CI)* | *p* |
| Intercept | 2.77  (1.73 – 4.61) | **<.001** | 5.60  (2.89 – 11.70) | **<.001** | 5.86  (2.99 – 12.48) | **<.001** |
| Study  (1 = Experiment 3) | 0.25  (0.13 – 0.49) | **<.001** | 0.24  (0.11 – 0.51) | **<.001** | 0.25  (0.11 – 0.53) | **<.001** |
| Andrea Knows  (1 = Yes) |  |  | 0.27  (0.13 – 0.55) | **<.001** | 0.27  (0.13 – 0.55) | **<.001** |
| Age  (years, scaled) |  |  | 0.98  (0.67 – 1.44) | .906 | 1.12  (0.68 – 1.87) | .667 |
| Study * Age |  |  |  |  | 0.72  (0.32 – 1.57) | .409 |
| Observations | 158 | | 158 | | 158 | |
| AIC | 201.702 | | 191.899 | | 193.211 | |
